# Generation and detection of orbital angular momentum via metasurface

**DOI:** 10.1038/srep24286

**Published:** 2016-04-07

**Authors:** Jinjin Jin, Jun Luo, Xiaohu Zhang, Hui Gao, Xiong Li, Mingbo Pu, Ping Gao, Zeyu Zhao, Xiangang Luo

**Affiliations:** 1 State Key Laboratory of Optical Technologies on Nano-Fabrication and Micro-Engineering, Institute of Optics and Electronics, Chinese Academy of Science, P.O. Box 350, Chengdu 610209, China; 2 University of Chinese Academy of Sciences, Beijing 100049, China; 3 Key Laboratory of Optoelectronic Technology and System, Ministry of Education, Chongqing University, Chongqing 400030, China

## Abstract

Beams carrying orbital angular momentum possess a significant potential for modern optical technologies ranging from classical and quantum communication to optical manipulation. In this paper, we theoretically design and experimentally demonstrate an ultracompact array of elliptical nanoholes, which could convert the circularly polarized light into the cross-polarized vortex beam. To measure the topological charges of orbital angular momentum in a simple manner, another elliptical nanoholes array is designed to generate reference beam as a reference light. This approach may provide a new way for the generation and detection of orbital angular momentum in a compact device.

The optical angular momentum of light can be separated into spin angular momentum (SAM), and orbital angular momentum (OAM) in the paraxial regime^1^. The SAM is associated with the polarization of the light, which has only two values of ±*ħ* (Plank’s constant divided by 2π) per photon. Beams with an azimuthal phase term *exp*(*ilφ*) possess an orbital angular momentum of *L* = *lħ* per photon, where *φ* is the azimuthal angle and *l* is topological charge as any integer ranging between −∞ and ∞. The special characterizes of OAM have attracted a great deal of interests from many realms, such as optical communications^2^,^3^,^4^, super-resolution imaging^5^, optical micromanipulation^6^, and detection of rotating objects^7^.

Typically, optical beams with OAM are generated using spiral phase plates^8^, spatial light modulators, computer generated holograms (CGH)^9^, birefringent elements^10^,^11^,^12^ and microscopic ring resonators^13^. Appearing in recent years, phase gradient metasurfaces have gained significant acceptance for their unique ability to flexibly manipulate the wavefront of light beam^14^,^15^,^16^,^17^,^18^,^19^,^20^,^21^,^22^,^23^,^24^,^25^,^26^,^27^,^28^. A typical structure of V-shape nanoantennas have been utilized to achieve the phase change of cross polarization light covering the whole range from 0 to 2π by varying the geometry of antennas when illuminated with linear polarization light^14^,^18^, in order to generate a new class of planar ultra-thin optical devices, such as deflector^14^,^15^,^16^, flat lens^17^, vortex plates^14^,^18^. Meanwhile, for circularly polarized light, a simpler metasurface has been used to control the wavefronts by rotating the orientation of antenna and then obtain the similar flat optical devices^21^,^22^,^23^,^24^,^25^,^26^,^27^,^28^. By comparison with the conventional method for generating OAM^8^,^9^,^10^,^11^,^12^, the approach with metasurfaces offer great advantages owing to their low profile and high degree of integration^14^,^18^,^26^,^27^,^28^. Furthermore, the efficiency, bandwidth and tunability can be boosted by combing the polarization controlling metasurface^29^,^30^.

On the other hand, determining the OAM state of an optical vortex has important applications in encoding information^31^, microscopy^32^, detection of rotating objects^7^, *etc.* Detecting the OAM state requires information of the phase distribution around the singularity, but direct measurement of the phase of far-field in the visible light is not possible. A more commonly used technique is to interfere the spiral wave front with a flat wave front generated by interferometer and count the number of spiral fringes^33^. In addition, cascading additional Mach-Zehnder interferometers and phase stepping techniques have been used to measure the OAM of a single photon^34^,^35^. One can also employ the diffraction pattern behind pointlike or triangular apertures to determine the OAM state of the incident light beams^36^,^37^. Alternatively, the diffractive optical elements such as the forked diffraction grating and the spatial light modulators (SLM) are used to check the state of OAM via state tomography^38^, transforming OAM states into transverse momentum states^39^ and phase flattening^40^. Obviously above detecting methods with the cascaded interferometric or the SLM require sub-wavelength experimental precision or maintains technically demanding for inclusion into larger systems.

In this paper, we proposed an ultra-thin metasurface with elliptical nanohole arrays to generate vortex beam with different OAM. Varying the major axis orientations of elliptical nanohole can obtain the phase shift of cross-polarization transmission light from 0 to 2π for circularly polarization light illumination. To measure the topological charge of OAM in a simple way, the other elliptical nanoholes array with parabolic phase distributions producing spherical wavefront is surrounding the area of elliptical nanoholes array that can generate the vortex beam. The interference between the helical wavefront and the spherical wavefront occurs in output cross-polarization light when illuminated with circularly polarization light. The topological charge and the chirality can be easily recognized in an image plane. The flat ultra-thin metasurface with thickness less than λ/4 has potential application in compact integrated optical systems.

## Results

### Simulated results

**Figure 1:**
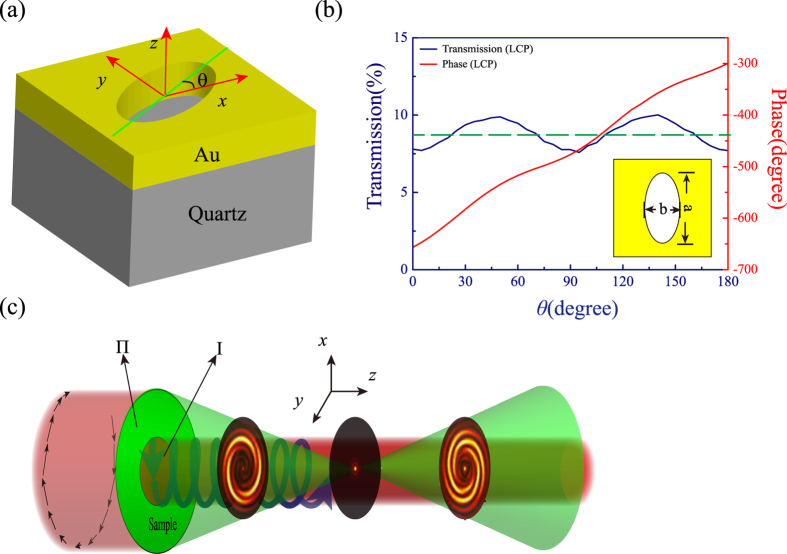
Schematic of basic unit cell in the simulation and interference pattern. (**a**)The unit cell of the elliptical nanohole milled in a thin gold film. The elliptical nanohole can be rotated in the *x-y* plane with an orientation angle *θ* to create a different phase delay. (**b**)The phase shifts and transmission of the cross-polarized light as the function of the orientation angle *θ* with a RCP incidence at wavelength of 632.8 nm. The long axis and short axis length of the nanohole in the simulation are *a* = 180 nm, *b* = 90 nm, respectively. (**c**) The sketch of the interference pattern. The regions I and II are used to generated vortex beam with OAM and spherical beam, respectively. The green pattern and red pattern behind the sample represent spherical beam and vortex light, respectively. The insert images are the interference intensity distribution. The polarization state of the incident light and transmitted light is presented by the space-variant arrows.

As shown in Fig. 1a, the elliptical nanoholes are selected as the components of phase control, which could convert a circularly polarized light into its opposite handedness. By changing the major axis orientations θ of elliptical nanohole, we can achieve approximate equal transmission and phase shift Φ covering range from 0 to 2π for the cross-polarization transmission light^8^,^21^,^23^. The transmission efficiency and phase of the cross-polarized light as a function of *θ* are plotted in Fig. 1b and the average cross-polarization transmission in the simulation is approximated to 8.7% (green dotted line). Note that the incident light in the simulation is right circular polarized light. The relationship between the phase change and the orientation of elliptical nanohole is given as Φ = ±2*θ,* where the signs of +/− correspond to the LCP/RCP incident light. According to the metasurface-assisted law of reflection and refraction^19^, arbitrary phase profiles Φ along the interface can be realized by rotating the major axis *θ.*

The schematic of generating and detecting the OAM with elliptical nanohole array is shown in Fig. 1(c). The metasurface is composed of two parts of elliptical nanohole arrays (the region I and II). One part (region I) is used to generate the OAM, and another (region II) as a flat lens that generate spherical wavefront. The interference between the helical wavefront and the spherical wavefront occurs in output cross-polarization light when illuminated with circularly polarization light in order to recognize the topological charge and the chirality in an image plane. To generate the OAM with elliptical nanohole array, the phase distribution of the vortex beam can be expressed as follows equation^21^,^22^.


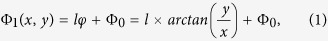


where *l* is the topological charge, *φ* is the azimuthally angle, and Φ_0_ is an arbitrary initial phase.

To measure the topological charge of OAM in a simple way, the other elliptical nanoholes array with parabolic phase distributions is designed to achieve a spherical beam as a reference light. The interference fringes generated by the spherical beam and vortex beam can reflect the phase distribution of the vortex beam. And the rotation direction of the interference fringes reveals the chirality of optical vortex. The spherical phase distribution can be expressed as follows:


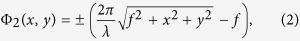


where *λ* is the free space wavelength and *f* is the focal length, the signs of +/− corresponding to the divergence and focusing of light.

According to the above phase distribution, the complex amplitudes of the electric fields of vortex beam and spherical beam can be expressed as *A*_1_exp(iΦ_1_) and *A*_2_exp(iΦ_2_), respectively. Thus the intensity distribution of the interference can be expressed as:


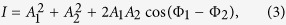


where 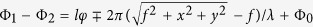.

**Figure 2:**
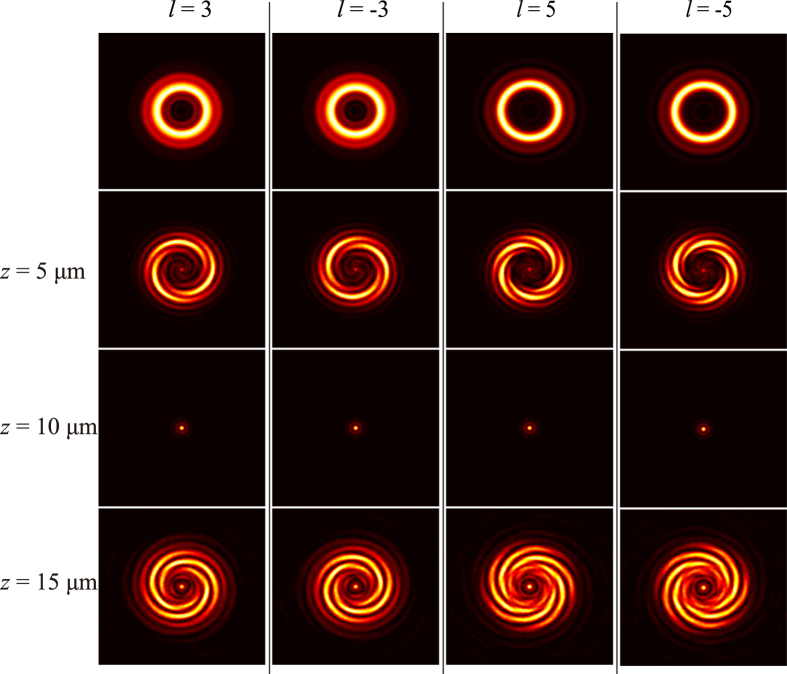
Numerical results for *l* = +3, −3, +5, −5, respectively. The first row shows intensity distribution of the vortex beam that without interference. The interference results at the position of *z* = 5 μm (before the focal plane), *z* = 10 μm (at the focal length plane) and z = 15 μm (after the focal plane), are shown in second, third and fourth row respectively.

The interference between the helical wavefront and the spherical wavefront occurs in output cross-polarization light resulting in spiral fringes, and the numbers of spiral lobes reveal the value of OAM. In addition, the sign of the topological charge can be distinguished from the helices of spiral fringes (the directions of counter-clockwise and clockwise indicate the sign of + and −, respectively). On the other hand, the opposite helices of the spiral lobes can be obtained when the reference beam is divergent as going away from the focal spot. The simulated results have been obtained by performing vectorial diffraction theory calculations^26^. In our simulations, we assume that the electric field in the structure area is the left circular polarized light with wavelength of 632.8 nm along the +*z* direction. The radius of the center area in Fig. 1c (region I with spiral phase distribution) is 5 μm, and the outer radius of concentric circles (region II with parabolic phase distribution) is 10 μm. The focal length of the spherical beam in the calculation is 10 μm. The electric field distributions with different topological charge of OAM (*l* = + 3, −3, + 5, −5) are plotted in Fig. 2. The ring-shape intensity distributions of vortex beam (the first row) show that it is difficult to recognize the topological charge of OAM without interference. When the vortex beam is interfered by the spherical beam, we can achieve the spiral fringes intensity distributions at the output plane *z* = 5 μm as shown in the second row (Fig. 2). We can count the number of fringes to determine the topological charge of OAM and resort to the rotation direction of spiral fringes to distinguish the sign of topological charge. At the focusing plane *z* = 10 μm, we can observe a spot image as shown in the third row (Fig. 2) because the intensity of focal spot is much larger than the intensity of vortex beam. For the image plane *z* = 15 μm beyond the focusing plane, the interference between the helical wavefront and the divergence wavefront could come into being as shown in the last row (Fig. 2). The spiral fringes can be observed clearly, with the rotation direction of spiral fringes being opposite to the case of convergence wavefront interference (*z* = 5 μm).

### Experimental results

**Figure 3:**
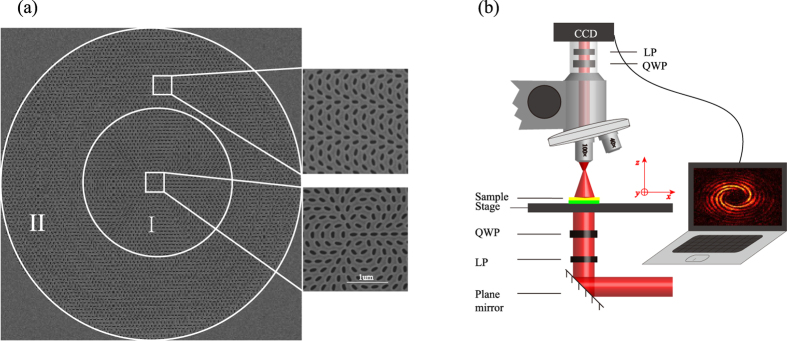
Schematic of nanohole arrays and the experimental setup. (**a**) SEM images of the nanohole arrays. The nanoholes array that can generate vortex beam with OAM is located at the center circle (region I), the outside ring (region II) is arranged to generate spherical beam. The insert show the arrangement of the elliptical nanoholes at different areas. (**b**) The schematic of the experimental setup for measuring the light intensity distribution that transmitted from the sample. The insert shows the interference results. Abbreviations for the optical components: QWP: quarter waveplate; LP: linear polarizer.

To further validate our approach, we fabricated and characterized the metasurface with topological charges of OAM *l* = −3and *l* = 5 as the scanning electron microscopy (SEM) image illustrated in Fig. 3a. The fabricated sample consists of two parts of elliptical nanohole arrays (the region I and II). The center area with spiral phase distribution (region I radius of 5 μm) could generate vortex beam, and the ring area with parabolic phase distributions (region II with inner radius of 5 μm and outer radius of 10 μm) could form the spherical wavefront. Note that the focal length of the flat lens (region II) is 10 μm and the fabricated samples with topological charges of OAM *l* = −3and *l* = 5 have identical sizes of geometry.

**Figure 4:**
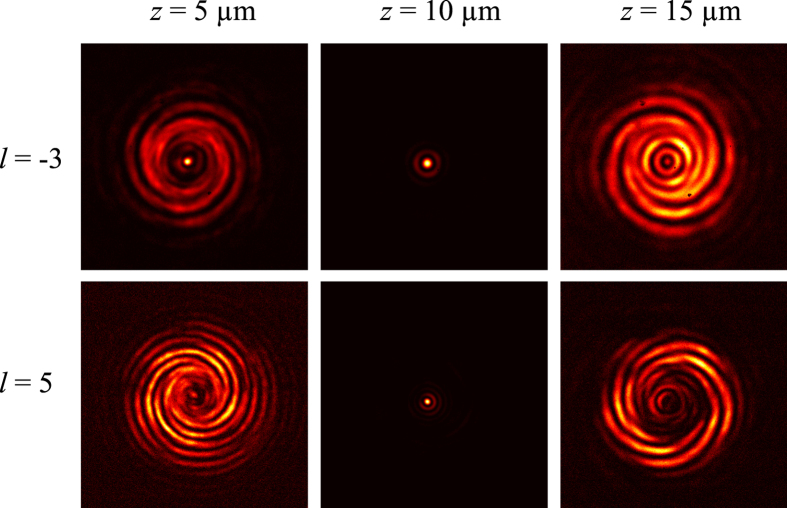
Measurement of the OAM beam. Images from left to right correspond to interfere with focal spherical light, focal plane and interfere with divergent spherical light, respectively. The number of the lobes of the helices and their rotation direction reveal that the transmitted beams possess OAM of −3 and 5, respectively. The measured patterns show excellent agreement with the calculated ones.

The OAM state generation is confirmed by the transmission measurement of right circularly polarized light at 632.8 nm. The schematic of the experimental setup is shown in Fig. 3b. We can obtain the intensity of cross-polarized transmission light (LCP) through placing a quarter wave plate and a linear polarizer before the CCD, and can also get the intensity images at different distances from the metasurface on the transmission side by changing the height of the stage. The experiment results that the cross-sectional planes (*x-y*) intensity distribution images at the different positions (*z* = 5 μm, *z* = 10 μm, *z* = 15 μm (from left to right)) are shown in Fig. 4. In the middle column of Fig. 4, a spot image appeared at the focal length plane *z* = 10 μm. Before the focal plane (*z* = 5 μm), the vortex beam interfered with the focusing spherical wavefront, resulting in the helix of the lobes with clockwise (*l* = −3) and counter-clockwise (*l* = 5). While, the opposite rotation direction of helix fringes emerged at the plane *z* = 15 μm due to the interference between the vortex beam and the divergent light after the focal plane. By counting the numbers of helix fringe and observing its rotation direction, we confirm that the generated vortex beam carries OAM value of *l* = −3 and 5. The measured patterns show excellent agreement with the calculated ones (Fig. 2). The conversion efficiency of our metasurface is on the order of 3%, which was defined by the ratio between the power of the cross-polarized light (LCP) transmitted through the sample and the incident power. Compared with the theoretical conversion efficiency, the reduction may originate from the reflection, absorption and the errors in the experiment.

## Discussion

**Figure 5:**
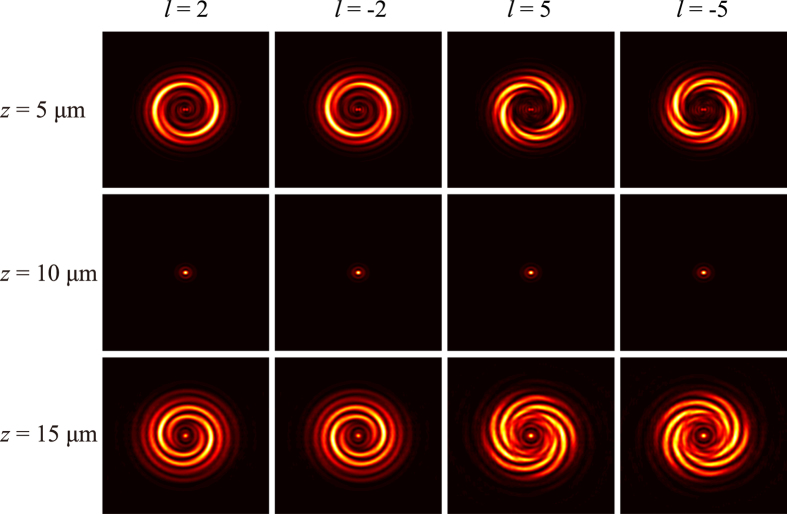
Numerical results with linear polarized incident light for *l* = +2, −2, +5, −5. The interference results at the position of *z* = 5 μm (before the focal plane), *z* = 10 μm (at the focal length plane) and *z* = 15 μm (after the focal plane), are shown in first, second, and third row, respectively.

In conclusion, we proposed the ultra-thin metasurface with array of elliptical nanoholes, which can realize the generation and measurement of the vortex beam for the circular polarization light illumination. Elliptical nanoholes array with spiral phase distributions could generate the vortex beam with arbitrary topological charges. Additional nanoholes array with parabolic phase distributions is designed on the metasurface to form a spherical beam as a reference light. We could recognize the topological charge of vortex beam via the interference fringes generated by the spherical beam and vortex beam. The refs 13 and 17 have demonstrated that the V-shaped nanotennas are arrayed to obtain the cross-polarization carrying the OAM under the linear polarization light illumination. The OAM is measured by the interference between the OAM beam and a reference beam generated by additional optical elements such as beam splitter. It is noted that our method is suitable for detecting the topological charge of OAM^14^,^18^ when designing another V-shaped nanotennas array with parabolic phase distributions is surrounding the area of V-shaped nanotennas array with spiral phase distribution. The interference between the helical wavefront and the spherical wavefront occurs in output cross-polarization light when illuminated with linearly polarization light. So the topological charge and the chirality can be easily recognized in an image plane and the results of numerical simulation with different topological charge are demonstrated in Fig. 5. In comparison with the previous measurements, our method can validly measure the OAM in a simple way to avoid the redundant components mentioned above. It is believed that this approach may provide a new way for the generation and detection of orbital angular momentum in a compact device.

## Methods

### Simulation

The finite element method in the commercial electromagnetic simulation software CST Microwave Studio was used to simulate the unit cell. A right circularly polarized plane wave is used as the incident light, and two Floquet ports in the +*z* and −*z* directions are considered. The propagation of the vortex and spherical beam were performed by vectorial diffraction theory calculations^26^. In the calculation, the light propagation was assumed along the positive direction of *z*, the electric field at *z* = 0 is known, the light field in *z* > 0 can be accurately determined.

### Fabrication

The fabrication began with depositing 3 nm thick chromium (Cr) onto a clean and planar quartz substrate to improve the adhesion between the gold film and substrate. Then a 120 nm thick gold film was deposited on the quartz substrate by magnetron sputtering. The elliptical nanoholes were then milled on the gold film using focused ion beam (FIB). The radius of the pattern area is 10 μm.

### Measurement

The custom-built microscope test system is used to experimentally characterize the performance of the sample. A HeNe laser with wavelength of 632.8 nm was used as the light source. First, the incident laser was converted into RCP light via the combination of a polarizer and a quarter-wave plate before illuminating onto the sample. The transmitted light passes through a 100 × objective lens and a tube lens, and collected by a silicon-based charge-coupled device (CCD) camera. For the measurement of cross-polarized component, the transmitted light was converted into linearly polarization by another quarter-wave plate and filtered by polarizer with polarization direction orthogonal to the first polarizer before the CCD camera aiming to filter the co-polarized component. The intensity images at different positions could be obtained by change the height of the stage. To ensure the precision of the experiment, the step length of the stage is 0.5 μm. The focal point of the 100X objective and the surface of the device are coincident at *z* = 0.

## Additional Information

**How to cite this article**: Jin, J. *et al*. Generation and detection of orbital angular momentum via metasurface. *Sci. Rep.* **6**, 24286; doi: 10.1038/srep24286 (2016).
